# Calculation of the contribution rate of China’s hydraulic science and technology based on a feedforward neural network

**DOI:** 10.1371/journal.pone.0222091

**Published:** 2019-09-11

**Authors:** Rongrong Xu, Yongxiang Wu, Ming Chen, Xuan Zhang, Wei Wu, Long Tan, Gaoxu Wang, Yi Xu, Bing Yan, Yuedong Xia

**Affiliations:** 1 Hydrology and Water Resources Department, Nanjing Hydraulic Research Institute, Nanjing, China; 2 State Key Laboratory of Hydrology-Water Resources and Hydraulic Engineer, Nanjing, China; 3 Hydrology and Water Resources College, Hohai University, Nanjing, China; 4 Sina Com Technology (China) Co. LTD, Beijing, China; 5 Water Conservancy Bureau of Pinghu, Jiaxing, China; Shandong University of Science and Technology, CHINA

## Abstract

Quantitative analysis of the contribution rate of China’s hydraulic science and technology and analysis of the underlying reasons behind changes provide an important foundation upon which the government can formulate water policies. This paper abandons the assumption of a scale economy and separates the changes of benefits brought about by the scale from scientific and technological progress, thus changing the C-D production function from linear to nonlinear. Based on a feedforward neural network, it calculates the coefficient of the output elasticity, the economic contribution rate of China’s hydraulic science and technology and the scale economies for each year using relevant data from 1981 to 2016. The results show that (1) the average contribution rate of capital investment from 1981 to 2016 was 47.3%, and the average contribution rate of labor from 1981 to 2016 was 9.1%. It is not obvious that the significant increase in the labor force has contributed to the growth of China’s water conservancy industry. (2) The average contribution rate of scale economies in 1981–2016 was 26.7%, and the contribution rate of scale economies is negatively correlated with the capital contribution rate. (3) The average contribution rate of China’s hydraulic science and technology was 43.6% from 1981 to 2016, and the average contribution rate of the total factor productivity after removing scale economies from 1981 to 2016 was 16.9%. During the period of the 6th Five-Year Plan(1981~1985), the contribution rate of water conservancy science and technology was relatively high. Since that time, it has remained at 40%. In recent years, as water conservancy reforms in key areas have made positive progress, scientific and technological progress has increased the growth of water conservancy benefits annually.

## 1 Introduction

In recent years, water resources in China have faced practical problems of flooding, droughts, water quality pollution, and soil loss [1], and the government of China has stated that the proportion of research and development investment in these sectors of the economy will increase to more than 2.5% of GDP by 2020, while the contribution rate of science and technology will reach more than 60% [2]. Quantitative analysis of the contribution rate of China’s hydraulic science and technology can provide valuable information on the investment benefits of China’s water conservancy projects [3]. However, accurately and scientifically measuring the contribution rate of science and technology has been a relatively complex theoretical problem that has not been well solved [4]. In particular, the benefits of water conservancy projects are significantly affected by the climate of each year, so they have strong uncertainty [5]. This paper evaluates the total factor productivity of water conservancy science and technology in China over the past 40 years.

The total factor productivity, as an important concept by which to measure economic efficiency, is an important tool for use in analyzing the sources of economic growth [6,7]. In particular, it is an important basis upon which the government can formulate policies for long-term sustainable development [8]. Many countries have proposed various methods to measure the total factor productivity, including the Cobb–Douglas production function [9], the Solow residual model [10], data envelopment analysis [11], and the frontier production function [12]. Cobb and Douglas [13] established mathematical models of inputs and outputs and calculated the contribution of technological progress to the increase in the total output value. Abramovitz [14] stated that in addition to the growth in outputs from production factors, other factors also contribute to total output value growth. Solow [15] proposed a production function that incorporates scientific and technological progress and quantitatively separates the role of scientific and technological progress in economic growth, Jorgenson [16] broke down production factors into capital and labor and further improved the Solow model.

Since the mid-1980s, a group of economists including Paul M.Romer and Lucas [17] have begun to break through the analysis framework of neoclassical growth theory and put forward new ideas on economic growth. Romer [18] constructed a model to explain the economic growth with increasing returns with scale and the externality of knowledge, proving that under the above assumptions, the economy may have a competitive equilibrium and that the social optimal competitive equilibrium is generally socially suboptimal. Lucas [19] put forward the assumption of perfect competition and began to build models under the framework of monopoly competition.

The total factor productivity has quite extensive applications in economics. It can be used to calculate the impacts of different inputs in an economy on the total factor productivity. Wu et al. [20] established a dynamic spatial regression model using provincial panel data and found that corruption directly reduces the total factor productivity of a region. Christiano et al. [21] estimated a New Keynesian model supposed that a firm is faced with price rigidity and found that the decline of the total factor productivity and rising operating costs played big roles during the Great Depression. The concept of total factor productivity has also been applied to climate change [22,23]. Zhong et al. [24] used data envelopment analysis to build a data model embedded with climate change information and calculated the total factor productivity of Chinese agriculture. Moore et al. [25] implemented empirical estimates of the temperature effects on GDP growth rates in the DICE model through two pathways: total factor productivity growth and capital depreciation. Dellink et al. [26] developed storylines of the shared socioeconomic pathways (SSPs), and predicted the factors affecting the GDP per capita, which are the total factor productivity, population, capital and energy. The concept of total factor productivity has also been applied to the study of carbon dioxide emissions [27,28]. Li et al. [29] used stochastic frontier functions to analyze the relationship between carbon dioxide and production technologies in China. Fan et al. [30] used the global Malmquist-Luenberger index method that consider carbon dioxide emission to study the improvement of the total factor CO_2_ emission performance relationship through scientific and technological progress. The production function and water price [31,32] have also been studied, and it has been shown that different production functions should be selected to better reflect reality [33].

The current method for measuring the contribution rate of water conservancy science and technology in a monopoly industry is mainly based on the C-D production function and the Solow residual value method [34,35]. Kong [36] used solow model to calculate the contribution rate of water conservancy science and technology in China, and predicted the future development of hydraulic technology [37]. The values of the capital-output elasticity coefficient α and the labor output elasticity coefficient β in the model strongly influence the final result of the contribution rate of water conservancy science and technology [38]. At present, there are some disadvantages to estimating model parameters using the empirical value method [39] or the neoclassical growth theory, the empirical value method is subjective, and the neoclassical growth theory lacks a solid foundation in reality [40]. The neoclassical growth theory has two core assumptions: one is exogenous technology, and the other is the constant return of the scale of production. Therefore, if there is a scale economy effect, the result of the water conservancy science and technology contribution rate will be distorted [41]. In this paper, we abandon the second assumption of the neoclassic growth theory, and separate the changes in benefits brought about by the scale from scientific and technological progress, thus changing the model from linear to nonlinear. Based on the partial derivative of the feedforward neural network output function, the partial derivative is used to fit the production function.

There are some limitations in using a multilayer feedforward network. The number of hidden layer nodes in neural network is related to the complexity of mapping relation. The fewer the number of hidden layer nodes, the more difficult the network is to describe complex problems. However, too much number will lead to the increase of training time of neural network, and too many networks will overfit. Therefore, when using neural network to calculate the contribution rate of water conservancy science and technology in China, it is necessary to choose the appropriate network structure to get the ideal result. Another defect of neural networks is local optimum, Most of the current algorithms can be divided into two categories: gradient descent and numerical computation. It is difficult to compare which algorithm is better in the actual situation, and researchers are often required to conduct simulation experiments.

Generally speaking, neural network is more complex than traditional solow residual method, and the network and algorithm need more elaborate design. However, through the neural network algorithm, the production function can be made more flexible, and the effect of economies of scale on the overall return can be well calculated. We abandon the unreasonable assumptions in the model and make the model closer to reality. In this paper, we design an algorithm to overcome. From this algorithm, the output elasticity of production factors is calculated, and the contribution rate of water conservancy science and technology is estimated for each year. The deep-seated reasons behind the changes are analyzed to provide a reference for the government to use when formulating water conservancy policies and long-term control measures.

This paper was organized as follows: Section 2 described the data and calculation method in detail. Section 3 set the key parameters of the neural network and described calculation results. Section 4 compared the results to those using another production function, and then discussed the development direction of water conservancy technology in China, summarized the concept and calculation method of the total factor productivity, and presented the future prospects for research on this topic.

## 2 Data and methodology

### 2.1 Data source and processing

#### 2.1.1 Calculation of the annual water conservancy benefits in China

The scope of the water conservancy benefits includes six major benefits: the irrigation benefit, hydropower benefit, flood control benefit, water supply benefit, soil and water conservation benefit, and water logging control benefit.

The irrigation benefit is mainly reflected in the increase in the yield and output of agricultural products due to irrigation. The increased agricultural output is the result of a combination of irrigation and agricultural measures and is deduced by the apportion coefficient of the water conservancy irrigation benefit.

The direct financial benefit of hydropower is obtained by multiplying the electric energy production (after deducting factory power consumption and line losses) by the electricity price. It is calculated by multiplying the hydroelectric energy production in China by the national average electricity price for water conservancy systems annually.

The benefits of new flood protection projects that reduce flood damage include the sum of the direct economic losses of various sectors of the national economy, including industry, agriculture, commerce, transportation, construction, and property in inundated areas. The flood control benefit is obtained by multiplying the area experiencing disaster reduction due to the flood prevention project by the total loss in terms of the Mu unit price.

The water supply benefit refers to the urban water supply benefits from water conservancy projects. After utilizing the input-output coefficient obtained from the weighted average of the water supply investment and water supply benefits over the years, the water supply benefits can be calculated according to the national annual investment in water supply projects.

The soil and water conservation benefits mainly include direct income from agriculture, forestry, animal husbandry, and fishing in addition to other social and economic benefits.

Waterlogging control benefits refer to the increased production of primary crops after building a waterlogging control project. This benefit generally reflects the national waterlogging control benefit, which is calculated according to the annual waterlogging control area and the waterlogging control benefit per acre.

The retail price index is selected for analysis, the economic benefits of China’s water conservancy system are shown in Table 1.

**Table 1:** Benefits from the water conservancy system from 1979 to 2016.

Year	Retail Price index	Flood control	Waterlogging control	Irrigation	Hydropower	Water supply	Soil and water conservation	Total
1979	94.34	213.69	92.66	196.58	50.37	2.55	270.38	826.23
1980	100.00	211.23	93.163	198.95	58.38	2.356	274.023	838.102
1981	102.40	187.22	93.417	197.778	65.736	3.146	277.319	824.616
1982	104.35	169.05	94.444	198.035	74.615	3.536	275.754	815.434
1983	105.91	146.11	95.007	197.558	86.608	7.418	282.366	815.067
1984	108.88	120.64	96.045	196.964	87.036	8.216	297.136	806.037
1985	118.46	108.01	97.008	195.062	92.642	8.448	308.922	810.092
1986	125.56	114.25	97.931	194.818	94.754	8.814	319.016	829.583
1987	134.73	118.49	98.961	195.2	100.52	8.97	329.797	851.938
1988	162.35	117.14	99.514	195.257	109.494	9.045	341.923	872.373
1989	192.39	116.49	100.377	196.709	118.643	9.154	347.283	888.656
1990	196.43	126.34	100.937	196.92	127.068	10.216	352.723	914.204
1991	214.67	141.42	102.209	199.207	125.162	11.86	371.814	951.672
1992	247.51	165.48	103.198	201.294	131.781	14.7	390.439	1006.89
1993	313.35	191.85	103.792	202.821	151.137	18.267	407.871	1075.74
1994	345.93	229.93	102.722	203.223	185.336	25.337	426.696	1173.24
1995	397.13	269.47	104.69	205.154	187.342	29.48	445.174	1241.31
1996	421.35	310.17	105.855	208.198	187.442	33.759	461.595	1307.02
1997	424.72	363.12	107.145	212.707	195.165	42.239	481.045	1401.42
1998	413.68	464.96	107.953	217.311	204.893	50.513	499.556	1545.19
1999	401.27	566.52	108.777	221.242	81.487	59.072	518.241	1555.34
2000	395.25	693.55	109.566	223.876	94.775	71.313	539.103	1732.18
2001	392.09	620	109.731	225.926	104.109	95.329	542.952	1698.05
2002	386.99	955.69	110.127	227.314	113.688	140.479	568.728	2116.03
2003	386.60	1037.09	110.337	227.487	121.119	176.41	657.317	2329.76
2004	397.43	1107.14	110.654	228.918	123.257	215.373	612.636	2397.98
2005	400.61	1113.5	111.395	230.181	151.739	250.084	630.282	2487.18
2006	404.62	1162.01	111.584	232.281	164.075	312.116	649.173	2631.24
2007	419.99	1189.61	111.808	235.145	180.422	401.836	665.021	2783.84
2008	444.77	1470.79	111.836	237.951	222.744	506.475	676.448	3226.24
2009	439.43	1600.16	112.67	241.165	200.58	690.521	696.144	3541.24
2010	453.06	2479.34	113.231	245.585	270.282	909.856	711.16	4729.45
2011	475.26	506	113.387	251.013	258.147	1152.65	730.231	3011.43
2012	484.76	892	114.095	254.305	343.494	1533.1	685.543	3822.53
2013	491.55	2358	114.543	258.303	369.168	1849.29	711.772	5661.08
2014	498.92	379	116.768	262.646	422.331	2155.86	743.182	4079.79
2015	506.91	422	118.562	268.07	441.426	2962.66	769.091	4981.81
2016	517.55	2354	120.41	274.423	466.546	3732.8	801.72	7749.89

unit: 100 million yuan

#### 2.1.2 Input data estimation

**Capital stock estimation**: This paper selects the fixed capital formation from the "China Water Conservancy Statistical Yearbook" as the investment flow indicator in the current year. Assuming that the output and capital stock have the same average growth rate over a given period, the average growth rate of the capital stock is $\text{g}\sqrt[{10}]{Y_{1990}/Y_{1980}}=0.0665$; *I*_1980_ = 2.707 billion; the average asset depreciation $=I_{0}\times \frac{(1+g)g}{\tilde{I}}-g=14.86\%$; and the base period capital stock *K*_1980_ = *I*_1980_ / (*g* + *d*) = 12.58484 billion. The capital stock of the water conservancy industry in each year was converted to the 1980 price level, as shown in Table 2.

**Table 2:** Capital and labor input to the water conservancy system over time.

Year	Fixed asset investment price index	Fixed assets formation (current prices)	Fixed assets formation (1980 prices)	Capital stock of fixed assets (1980 prices)	Labor force
1980	100.00	27.07	27.07	125.85	102.49
1981	103.00	13.57	13.14	120.32	102.43
1982	106.00	17.48	16.54	118.93	102.315
1983	108.00	21.13	19.52	120.82	101.775
1984	113.00	20.68	18.36	121.17	104.18
1985	121.00	20.16	16.69	119.82	107.98
1986	129.00	22.86	17.79	119.74	111.15
1987	135.00	27.08	20.03	122.01	118.645
1988	154.00	30.65	19.96	123.78	128.805
1989	167.00	35.55	21.33	126.67	134.745
1990	180.36	48.72	27.72	134.86	136.88
1991	192.00	64.87	33.71	148.61	140.8
1992	222.00	97.17	43.80	170.29	144.25
1993	281.00	124.93	44.48	189.45	147.95
1994	310.00	168.74	54.42	215.73	149.98
1995	328.29	206.32	62.83	246.52	151.625
1996	341.42	238.51	69.84	279.74	156.55
1997	347.23	315.41	90.81	329.01	158.405
1998	346.53	467.56	134.89	415.05	155.705
1999	345.15	499.15	144.58	497.99	151.11
2000	348.94	612.94	175.61	599.65	143.455
2001	350.34	560.97	160.08	670.66	134.77
2002	351.04	819.22	233.31	804.37	130.16
2003	358.76	743.41	207.17	892.06	125.865
2004	378.85	790.31	208.55	968.10	120.525
2005	384.91	827.39	214.89	1039.20	114.33
2006	390.69	932.72	238.68	1123.51	109.815
2007	405.92	1026.52	252.81	1209.44	107.965
2008	442.05	1604.09	362.78	1392.59	106.165
2009	431.44	1702.69	394.55	1580.31	104.655
2010	446.97	2707.61	591.07	1951.25	105.215
2011	476.47	3452.1	732.39	2385.80	104.58
2012	481.81	4117.2	854.53	2885.80	102.935
2013	483.26	3954.0	818.19	3275.16	103.7
2014	485.67	4345.1	894.65	3683.126	100.55
2015	476.93	5452.2	1143.18	4278.99	95.9
2016	488.85	6113.99	1250.69	4929.77	93.6

Unit: 100 million yuan, 10,000 persons

**Labor input estimation**: The contribution of labor to the water conservancy system, excluding labor by those engaged in work with earth and stone, should be attributed to the labor input. According to the relevant data from the "China Water Conservancy Statistical Yearbook" for past years, the annual labor input of the national water conservancy system is calculated by taking the average between the beginning and ending dates of the year, as shown in Table 2.

### 2.2 Production function selection

Among the many production functions, the most commonly used production functions are the Cobb-Douglas production function, linear production function, Leontief production function[42], constant elasticity of substitution production function[43] and translog production function[44], as shown in Table 3.

**Table 3:** Commonly used form of the production function.

Production function name	Production function form
Cobb-Douglas production function	$\text{y}=\beta_{0}\prod_{n=1}^{N}x_{n}^{\beta_{n}}$
Linear production function	$\text{y}=\beta_{0}+\sum_{n=1}^{N}\beta_{n}x_{n}$
Leontief production function	$\text{y}=\sum_{n=1}^{N}\sum_{m=1}^{M}\beta_{nm}{\left(x_{n}x_{m}\right)}^{0.5}$
Constant elasticity of substitution production function(CES)	y=β0(∑n=1Nβnxnρ)mρ
Translog production function	$\text{y}=\text{exp}(\beta_{0}+\sum_{n=1}^{N}\beta_{n}Inx_{n}+\frac{1}{2}\sum_{n=1}^{N}\sum_{m=1}^{M}\beta_{nm}lnx_{n}lnx_{m})$

The main differences between the production functions lie in the assumption of elasticity of substitution, and the values of the elasticity of substitution of these production functions are shown in Table 4.

**Table 4:** Commonly used forms of production functions.

Production function name	Elasticity of substitution
Cobb-Douglas production function	1
Linear production function	∞
Leontief production function	0
Constant elasticity of substitution production function	Constant
Translog production function	Variable

According to the characteristics of each production function, we can obtain some conclusions:

1. The linear production function describes a production model with a constant return on scale, which can be used in economic analysis, including constant economies of scale. In this paper, we abandon the assumption that the economies of scale of the CD production function are constant.
2. The constant elasticity of the substitution production function (CES) improves the elasticity of substitution of the Cobb-Douglas production function. For different research objects or different sample intervals of the same research object, the elasticity of substitution is different because of the difference in sample values. The substitution elasticity of the constant elasticity of the substitution production function is $\frac{1}{1+\rho }$, while the substitution elasticity of the Cobb-Douglas production function is 1, and this makes the constant elasticity of the substitution production function more realistic than that of Cobb-Douglas production function.
3. The elasticity of substitution of the Leontief production function is zero, and this shows that there is no substitution between input factors.
4. The translog production function can be converted to any form of second-order Taylor approximation of the production function, and it can be used to test whether the elasticity of substitution is constant. The translog production function has good mathematical properties, but it requires complete panel data, so the transcendental logarithm function is not used for comparison in this paper.

Based on the data in this article, we used a more general C-D production function that considers scale economy effects for analysis, and we also used CES production functions to compare the results of the C-D production function. The methods of CES production functions are shown in the S1 File.

The C-D production function and Solow residual value method assume that the sum of the output elasticities of production factors is 1. If there is no scale economy, this assumption will attribute the changes in benefits brought about by the scale change to scientific and technological progress. The C-D production function and Solow residual method do not consider the impacts of scale economies, which will lead to errors in the results of the contribution rate of scientific and technological progress.

This paper does not assume scale economies. The production function:

$$\text{Q}(\text{t})={\text{A}}_{t}K^{\alpha }L^{\beta }$$ (1)

Let capital productivity *P*_1_ = *Y*/*K* and labor productivity *P*_2_ = *Y*/*L*, Convert Eq (1) to:

$$\text{Q}(\text{t})=P_{1}^{\alpha^{*}}P_{2}^{\beta^{*}}K^{\alpha^{*}}L^{\beta^{*}}$$ (2)

In Eq (2), α* = *α* / (*α* + *β*), β* = *β* / (*α* + *β*), α* + β* = 1.

Let $\text{y}=\frac{\Delta Q(t)}{Q(t)},\;\text{k}=\frac{\Delta K}{K},\;\text{l}=\frac{\Delta L}{L},\;\text{a}=\frac{dlnA_{t}}{dt}$. Change Eq (1) to a differential form:

$$\begin{array}{c} {\text{y}}_{t}=a_{t}+\alpha_{t}k_{t}+\beta_{t}l_{t}=a_{t}+\left[\left(\alpha_{t}-\alpha_{t-1}\right)k_{t}+\left(\beta_{t}-\beta_{t-1}\right)l_{t}\right]+a_{t-1}k_{t}+\beta_{t-1}l_{t}\\ =a_{t}+\left[\left(\alpha_{t}-\alpha_{t-1}\right)k_{t}+\left(\beta_{t}-\beta_{t-1}\right)l_{t}+\left(\alpha_{t-1}-\alpha_{t-1}^{*}\right)k_{t}+\left(\beta_{t-1}-\beta_{t-1}^{*}\right)l_{t}\right]+\left(\alpha_{t-1}^{*}k_{t}+\beta_{t-1}^{*}l_{t}\right)\\ =a_{t}+\left[\left(\alpha_{t}-\alpha_{t-1}^{*}\right)k_{t}+\left(\beta_{t}-\beta_{t-1}^{*}\right)l_{t}\right]+\left(\alpha_{t-1}^{*}k_{t}+\beta_{t-1}^{*}l_{t}\right). \end{array}$$ (3)

*α*_*t*_ and *β*_*t*_ are the output elasticities of capital and labor in year t. The first item in Eq (3) is the Solow residual value produced after eliminating the scale economy. The second item is the output change (*s*_*t*_) brought about by the scale economy effect. The sum of the first two items constitutes the total factor productivity (TFP). The third item is the output changes brought about by the change in production factors. Through this method, the increased output brought by scale economies can be removed from the TFP.

### 2.3 The C-D production function calculation method

#### 2.3.1 The challenges of the C-D production function calculation in a multilayer feedforward network

Next, the effect of the scale economy on the output growth must be calculated based on the Eq (3). When using multivariate regression analysis, which requires a longer time series, it is easy to miss changes in capital, labor, and technology for the current year. Additionally, in this paper, if α and β are constants, the output change (*s*_*t*_) brought about by the scale economy effect will be equal to zero, which will be contrary to the original intention of this paper. Therefore, ordinary least squares(OLS) cannot be used.

A multilayer feedforward network can describe extremely complex nonlinear systems and handle the random characteristics of the original data. A multilayer feedforward network is a universal approximator that can automatically simulate a system when there is sufficient training time.

In the structural design of a feedforward network, the node scale should be reduced as much as possible to reduce the system complexity and learning time while still meeting the requirements. The number of nodes is related to the complexity of the object to be expressed in the model. If the number is too small, the model struggles to describe real problems. If the number is too large, however, the degree of nonlinearity becomes higher, the training time increases, and the model becomes prone to the “overlearning” phenomenon. As a result, the ability of the model to generalize deteriorates. Therefore, the principle used to select the number of nodes in this paper is based on the correct reflection of the relationship between the input and output, and fewer hidden layer nodes are used to make the network structure as simple as possible.

The multilayer feedforward network uses a single-track multilayer structure in which each layer contains several nodes. The nodes in the same layer are not connected and the information between the layers is transmitted in only one direction. A single-layer feedforward network can only distinguish linearly separable modes while a multilayer feedforward network can be used for any classification problem. In addition, a multilayer feedforward network can be used as a general function approximator. A two-layer feedforward network can approximate any complex continuous function as long as there are enough nodes in its hidden layer with an s-shaped activation function of the hidden layer and a linear activation function of the output node. Therefore, this paper selects a two-layer feedforward network with a hidden layer as the research method.

In the research process, we find that there are two main problems of the BP algorithm: the slow convergence speed and the local minimum point of the function. To resolve the slow convergence speed, a network pruning algorithm is used to delete redundant input nodes. At the same time, based on the sensitivity analysis, we analyze the influence of parameters on the system, the influence of small disturbances in the parameters on the network output, and the importance of the network parameters. When the function has a local minimum point, the adaptive variable step length algorithm is selected, which means that the learning compensation adjusts with the variation in the error surface.

#### 2.3.2 The solutions of the C-D production function calculation

**1) Sensitivity Analysis**

Sensitivity analysis is also called perturbation analysis. It is an analytical method used to determine the sensitive factors and their degree of influence.

The sensitivity function *S* (*y*_*i*_, *x*_*j*_) indicates how the change in the system input *x*_*j*_ affects the system output *y*_*i*_. There are four calculation methods, as follows:

Normalized sensitivity:

$$\text{S}(y_{i},\;x_{j})=\frac{\partial y_{i}/y_{i}}{\partial x_{j}/x_{j}}=\frac{x_{j}}{y_{i}}\frac{\partial y_{i}}{\partial x_{j}}$$ (4)

Seminormalized sensitivity by *y*_*i*_:

$$\text{S}({\text{y}}_{\text{i}},\;{\text{x}}_{\text{j}})=\frac{\partial {\text{y}}_{\text{i}}}{\partial {\text{x}}_{\text{j}}/{\text{x}}_{\text{j}}}={\text{x}}_{\text{j}}\frac{\partial {\text{y}}_{\text{i}}}{\partial {\text{x}}_{\text{j}}}$$ (5)

Seminormalized sensitivity by *x*_*i*_:

$$\text{S}({\text{y}}_{\text{i}},\;{\text{x}}_{\text{j}})=\frac{\partial {\text{y}}_{\text{i}}/{\text{y}}_{\text{i}}}{\partial {\text{x}}_{\text{j}}}=\frac{1}{{\text{y}}_{\text{i}}}\frac{\partial {\text{y}}_{\text{i}}}{\partial {\text{x}}_{\text{j}}}$$ (6)

Unnormalized sensitivity:

$$\text{S}({\text{y}}_{\text{i}},\;{\text{x}}_{\text{j}})=\frac{\partial {\text{y}}_{\text{i}}}{\partial {\text{x}}_{\text{j}}}$$ (7)

**2) Configuration Optimizing Theory**

Sensitivity analysis is used to define the sensitivity of nodes, and then the relative total sensitivity is defined. The relative total sensitivity index is used to characterize the influence degree of the parameters on the model output. The input node, hidden layer node, and connection weight are selected using the relative total sensitivity index, and then the parameters with little or no effect can be deleted.

**Definition 1** For mode *u*, ${\text{x}}_{\text{j}}^{\left(\text{u}\right)}$ represents the input of the *j* th input node, and $y_{i}^{\left(u\right)}$ represents the output of the *i* th output node. The sensitivity of the network output function F(***x*, *w***) with respect to the input node can be defined as:

$$s_{ij}^{\left(u\right)}=\frac{\partial y_{i}^{\left(u\right)}/y_{i}^{\left(u\right)}}{\partial x_{i}^{\left(u\right)}/y_{i}^{\left(u\right)}}=\frac{\partial y_{i}^{\left(u\right)}}{\partial x_{j}^{\left(u\right)}}\frac{x_{j}^{\left(u\right)}}{y_{i}^{\left(u\right)}}$$ (8)

This is referred to as the sensitivity of output *i* to input *j* for mode *u*. ${ts}_{j}^{\left(u\right)}=\sqrt{\sum {\left(s_{ij}^{\left(u\right)}\right)}^{2}}$ is the total sensitivity of the input vector to input *j*.

**Definition 2** For a given training set, $\text{A}S_{ij}=\sqrt{\sum {\left(s_{ij}^{\left(u\right)}\right)}^{2}/s}$ is the average sensitivity of output *i* to input *j*, and $RSI_{ij}=\frac{AS_{ij}}{\sum AS_{ij}}$ is the relative sensitivity index of output *i* to input *j*. The weight of each output variable in the system is not exactly the same: A*S*_*ij*_ must be weighted.

$$\text{T}S_{ij}=\sqrt{\sum {\left(\lambda_{ij}AS_{ij}\right)}^{2}}$$ (9)

*λ*_*ij*_ is the weight of *AS*_*ij*_, and satisfies ∑_*j*_ *λ*_*ij*_ = 1, *λ*_*ij*_ ≥ 0, *i* = 1, 2, …, *n*.

In the training process, some nodes have a strong network correlation but represent small proportions. The average sensitivity A*S*_*ij*_ calculation method may delete nodes. Further, by using the average calculation method, training will be reduced, but a stronger correlation will be suppressed. Therefore, we need to calculate the variance of the node to be deleted $\sigma_{j}^{2}=Var({\left({ts}_{j}^{\left(1\right)},{ts}_{j}^{\left(2\right)},\ldots ,{ts}_{j}^{\left(s\right)}\right)}^{T})$ to make further judgments.

**3) Sensitivity Calculation**

To carry out sensitivity analysis, $\frac{\partial y_{i}^{\left(u\right)}}{\partial x_{j}}$ needs to be calculated. Take the derivative of Eqs (1) ~ (3):

$$\frac{\partial y_{i}^{\left(u\right)}}{\partial x_{j}}=\sum_{k=1}^{l}f_{2}^{’}({\vec{y_{i}}}^{\left(u\right)})\cdot w_{ik}^{2}\cdot f_{1}^{’}({\vec{h_{i}}}^{\left(u\right)})\cdot w_{kj}^{1}$$ (10)

The training set instance ***x***^(*u*)^ can calculate the partial derivative of the network output function F(***x*, *w***) with respect to the input x. If u = l, 2, …, s, s partial derivative matrixes can be obtained by repeated calculations, and the elements in the matrix will be partial derivatives that are shaped like formula (10). If expressed by a matrix symbol, Eq (10) can be written as:

$$J_{x}^{\left(u\right)}=\Gamma_{2}^{\prime }W^{2}\Gamma_{1}^{\prime }W^{1}$$ (11)

$J_{x}^{\left(u\right)}$ is the Jacobian matrix of the network output function F(***x*, *w***) at ***x***^(*u*)^;

***W***^1^ is the connection weight matrix of the first layer;

***W***^2^ is the second-level connection weight matrix;

$\Gamma_{1}^{\prime }$ is a diagonal matrix $\text{diag}(f_{1}^{’}({\vec{h_{1}}}^{\left(u\right)}),f_{1}^{’}({\vec{h_{2}}}^{\left(u\right)}),\ldots ,f_{1}^{’}({\vec{h_{l}}}^{\left(u\right)}))$;

$\Gamma_{2}^{\prime }$ is a diagonal matrix $\text{diag}(f_{2}^{’}({\vec{y_{1}}}^{\left(u\right)}),f_{2}^{’}({\vec{y_{2}}}^{\left(u\right)}),\ldots ,f_{2}^{’}({\vec{y_{n}}}^{\left(u\right)}))$;

The sensitivity of the hidden layer node, relative sensitivity, and partial derivative can be similarly obtained.

**4) Calculation of the output elasticity coefficient**

The econometric model of a multilayer feedforward network can be expressed as:

$$y_{i}^{\left(t\right)}=f(x_{1}^{\left(t\right)},x_{2}^{\left(t\right)},\ldots ,x_{m}^{\left(t\right)})$$ (12)

It can be obtained from Eq (12):

$$\frac{dy_{i}^{\left(t\right)}}{y_{i}^{\left(t\right)}}=s_{i1}^{\left(t\right)}\frac{dx_{1}^{\left(t\right)}}{x_{1}^{\left(t\right)}}+s_{i2}^{\left(t\right)}\frac{dx_{2}^{\left(t\right)}}{x_{2}^{\left(t\right)}}+\cdots +s_{im}^{\left(t\right)}\frac{dx_{m}^{\left(t\right)}}{x_{m}^{\left(t\right)}}$$ (13)

$s_{ij}^{\left(t\right)}$ is defined as the Eq (4) normalized sensitivity.

Suppose that:

$$J^{\left(t\right)}=\left(\begin{array}{ccc} \frac{\partial y_{1}^{\left(t\right)}}{\partial x_{1}} & \cdots & \frac{\partial y_{1}^{\left(t\right)}}{\partial x_{m}}\\ \vdots & \ddots & \vdots \\ \frac{\partial y_{n}^{\left(t\right)}}{\partial x_{1}} & \cdots & \frac{\partial y_{n}^{\left(t\right)}}{\partial x_{m}} \end{array}\right)$$

$$dy^{\left(t\right)}={\left(dy_{1}^{\left(t\right)},dy_{2}^{\left(t\right)},\ldots ,dy_{n}^{\left(t\right)}\right)}^{T}$$

$$dx^{\left(t\right)}={\left(dx_{1}^{\left(t\right)},dx_{2}^{\left(t\right)},\ldots ,dx_{n}^{\left(t\right)}\right)}^{T}$$

$$dy^{\left(t\right)}=J^{\left(t\right)}dx^{\left(t\right)}$$ (14)

Eq (14) can be expressed as:

$$\frac{dy^{\left(t\right)}}{y^{\left(t\right)}}=S^{\left(t\right)}\frac{dx^{\left(t\right)}}{x^{\left(t\right)}}$$ (15)

***S***^(*t*)^ is a normalized sensitivity matrix.

In this paper, $y_{i}^{\left(t\right)}=f(x_{1}^{\left(t\right)},x_{2}^{\left(t\right)},\ldots ,x_{m}^{\left(t\right)})={\text{A}}_{t}K^{\alpha }L^{\beta }$, where A_*t*_, α, and β are parameters that change with time.

**5) Key parameters of production function fitting**

The input data are normalized to speed up the network convergence. Three datasets are selected as the test set, and the remaining 33 datasets are used as the training set. The initial network structure can be taken as n-l-m, and when the RTS*I*_*j*_ of a hidden layer node is smaller than $\text{a}=\frac{1}{10l}$ (where l is the number of hidden layer nodes), the node should be deleted. The training goal is to achieve a mean square error (MSE) of 10^−6^, which can ensure the robustness of the model. The code can be found in S2 File.

## 3 Results

### 3.1 Parameter settings of the neural network

This paper establishes a multilayer feedforward network model. After training MATLAB’s neural network toolbox, the output function is obtained in the form of Q(t) = A_*t*_*K*^*α*^*L*^*β*^.

The input data are normalized to speed up the network convergence. Three datasets are selected as the test set, and the remaining 33 datasets are used as the training set. The training goal is to achieve a mean squared error (MSE) of 10^−6^. The maximum number of cycles is 5000.

The initial network structure is taken as 5-10-1, and when the RTS*I*_*j*_ of a hidden layer node is smaller than $\text{a}=\frac{1}{10l}$ (where l is the number of hidden layer nodes), the node should be deleted. The network structure optimization algorithm is applied to obtain the simplest network structure 3-4-1. The MSEs of the training set and the test set are 1.0264×10^−6^ and 1.0057×10^−6^. The connection weight matrix and threshold are W^1^ = [-1.0245–2.9913] W^2^ = [-1.6944–9.2483] b^1^ = -0.1367 b^2^ = -6.9678.

When the multilayer feedforward network fits the production function, because the input and output data are normalized, the partial derivative needs to be multiplied by a corresponding coefficient to obtain the estimated value of the partial derivative of the potential function. Then the output elasticities α and β of the production factors are found, as shown in the S3 File.

To determine the contribution rate of scientific and technological progress, the following indicators need to be calculated.

First, the average annual growth rate y, k, l should be calculated. Because the scope of flood disaster reduction and the extent of disasters in water conservancy projects are also random variables affected by climatic factors, benefits such as flood control and water logging control show greater volatility interannually. Therefore, the horizontal method is used to calculate the output. Taking the output as an example: $\text{y}=\sqrt[{t}]{\frac{Y_{t}}{Y_{0}}}-1$, where *Y*_*t*_ is the output efficiency of the water system during period t.

According to Eq (3), the contribution rate of the Solow residual value to the total output growth rate *E*A_*t*_ is:

$$E{\text{A}}_{t}=\frac{a_{t}}{y_{t}}\times 100\%$$ (16)

The contribution of the scale economy to the total output growth *E*S_*t*_ is:

$$E{\text{S}}_{t}=\frac{s_{t}}{y_{t}}\times 100\%$$ (17)

The contribution of the capital input to the total output *E*K_*t*_ is:

$$E{\text{K}}_{t}=\frac{\alpha_{t-1}^{*}k_{t}}{y_{t}}\times 100\%$$ (18)

The contribution rate of the labor input to the total output *E*L_*t*_ is:

$$E{\text{L}}_{t}=\frac{\beta_{t-1}^{*}l_{t}}{y_{t}}\times 100\%$$ (19)

The calculation results are shown in the S3 File.

### 3.2 The growth of benefits of water conservancy according to the input factor

The contributions of different input factors to the economic benefits of water conservancy over the years show different trends. The water conservancy industry is a traditional infrastructure industry. The average contribution rate of capital input from 1981 to 2016 was 47.3%, and the average contribution rate of labor input from 1981 to 2016 was 9.1%.

During the process of reform and opening up, the scale of capital input was relatively small, but as time passed, the water conservancy investment gradually increased. From 1981 to 1993, the contribution rate of capital investment rose from 14.0% to 60.8%, and then it declined slowly. After the outbreak of the international financial crisis in 2008, the central government of China implemented a proactive fiscal policy and increased water conservancy infrastructure construction. As a result, the contribution rate of capital investment rose again and then declined, and the capital contribution rate was 37.1% in 2016.

**Fig 1:**
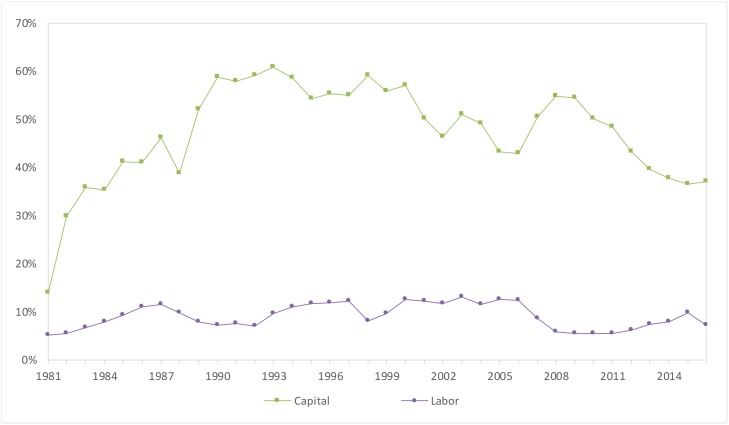
Trend of the input factor contribution rate.

As shown in Fig 1, the contribution rate of the labor input remained relatively stable between 1981 and 2016. It is not obvious that the significant increase in the labor force contributed to the growth of China’s water conservancy industry because laborers in China are not productive. Taking a long-term perspective, China’s economy must enter an intensive growth period and policy makers should pay attention to the utilization of human resources.

### 3.3 The growth of the benefits of water conservancy based on the scale economy

**Fig 2:**
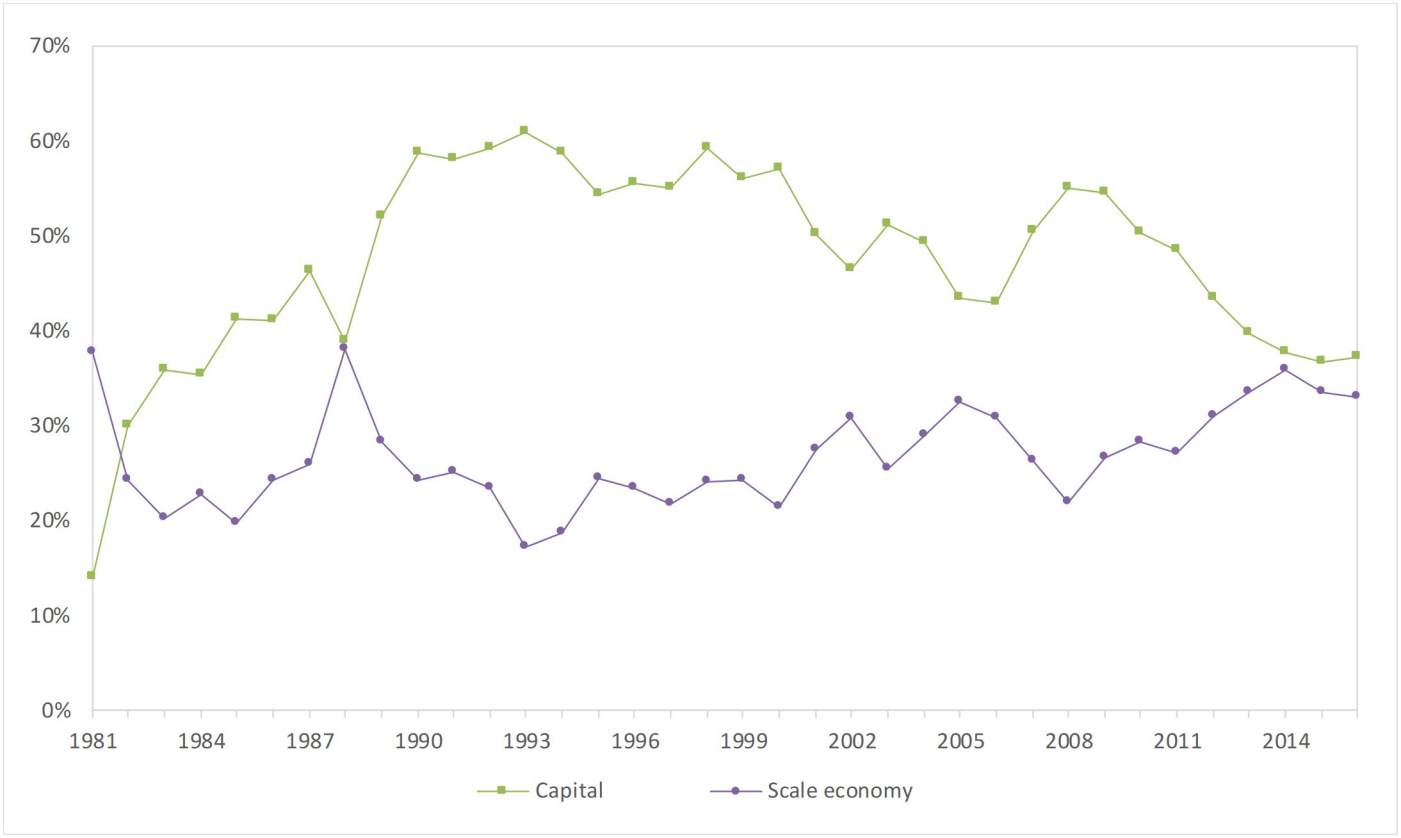
Trends of the scale economy and capital contribution rate.

The average contribution rate of the scale economy from 1981 to 2016 was 26.7%. As shown in Fig 2, the scale economy contribution rate has a negative correlation with the capital contribution rate.

From 1981 to 2007, the scale economy contribution rate was relatively stable. After the reform and opening up, the invisible hand of the market economy gradually played a role, and the scale economy contribution rate has increased; thus, the capital investment can bring multiple investment returns, and there has been less water conservancy industry duplicate construction during this period. After the implementation of the proactive fiscal policy in 2008, the central government of China implemented a large amount of investment in water conservancy construction, which led to a certain amount of duplication of construction, and the scale of the economic contribution declined and then returned to normal levels.

### 3.4 Scientific and technological progress in the growth of water conservancy

The average rate of the scientific and technical contribution to water conservancy from 1981 to 2016 was 43.6%. After excluding the economies of scale, the average contribution rate of the TFP was 16.9%.

During the period of the 6th Five-Year Plan (1981~1985), although the scale of capital investment was small, the national scientific and technological research plan within the 6th Five-Year Plan heavily subsidized water conservancy. The implementation of the “Study of the South-to-North Water Diversion Project,” “Technology development of large-scale hydropower stations”, “Studies on the Development and Utilization of Water Resources and Comprehensive Evaluation in East China” and other projects quickly compensated for technical problems in traditional infrastructure construction and became the main impetus promoting the growth of water conservancy benefits in the early stages of reform and opening up.

Since then, the contribution rate of water science and technology has remained at 40%. With the implementation of strict water resource management systems and the construction of large-scale water conservancy projects, water conservancy reforms in key areas have made positive progress. Scientific and technological progress has increased the growth of water conservancy benefits annually. In 2015, the contribution rate of science and technology was 53.6%, compared with 55.7% in 2016.

**Fig 3:**
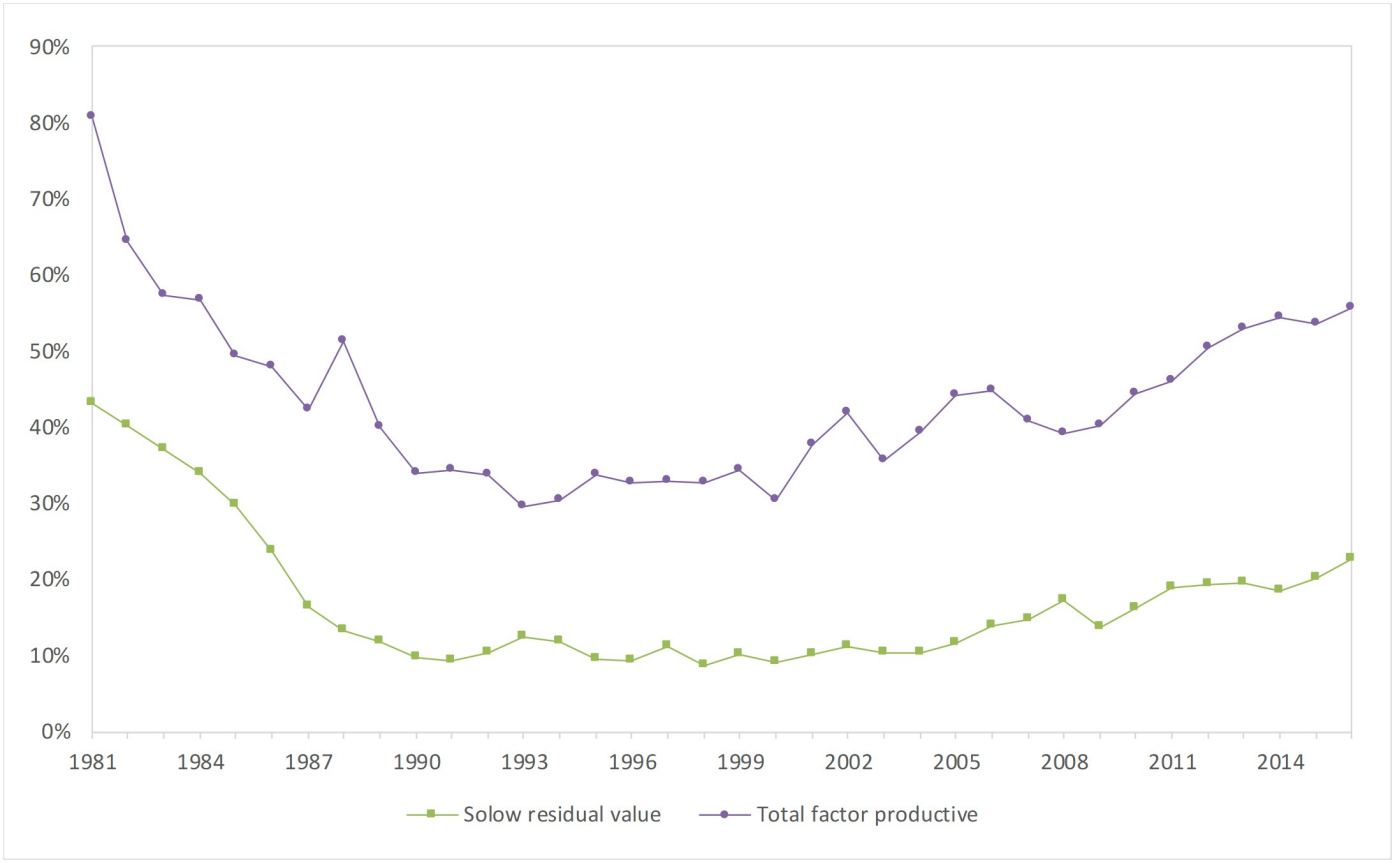
Trend of the contribution rate of scientific and technological progress.

As shown in Fig 3, Solow’s residual value has been decreasing since 1981. The growth of water conservancy emphasizes the formation of physical capital. If the capital allocation is not reasonable, the high capital accumulation rate will compensate for the economic operation inefficiency. The Chinese economy has shown extensive features over a period of time, but in the last ten years, the TFP has again increased, and economic growth has shifted from the extensive mode to the intensive mode.

## 4 Discussion

### 4.1 Comparison of the results of the CES production function

There is no research on the contribution rate of water conservancy science and technology in China, so we choose the calculation results of the CES production function for a comparative analysis.

As shown in the S3 File, *ρ* is approximately equal to zero, and this proves that selecting the Cobb-Douglas production function can basically meet the research needs. Then we compare the contribution rates of the capital, labor, total factor productivity, economies of scale and Solow residual value as calculated by the Cobb-Douglas production function and the constant elasticity of substitution(CES) production function.

**Fig 4:**
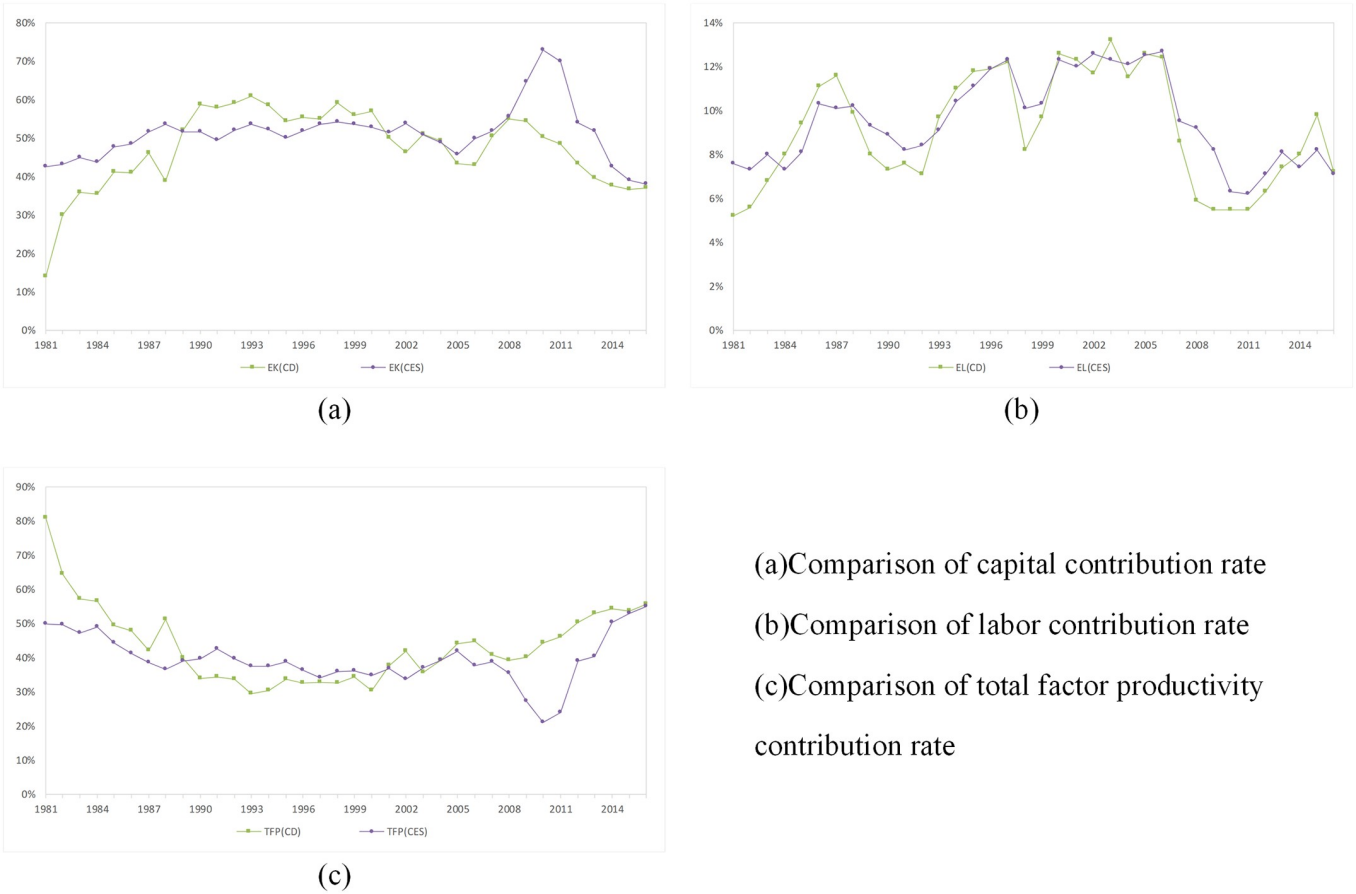
Comparison of the contribution rates calculated by the CES production function and the Cobb-Douglas production function.

As shown in Fig 4, the capital contribution rate of the CES production function is relatively stable compared with that of the Cobb-Douglas production function. However, the capital contribution rate of the CES production function from 2008 to 2011 was higher than that of Cobb-Douglas production function. The labor contribution rate of the CES production function is consistent with that of the Cobb-Douglas production function. The total factor productivity of the CES production function has the same trend as that of the Cobb-Douglas production function, but it is lower than that of the Cobb-Douglas production function from 2008 to 2011. The contribution rates of capital and the total factor productivity as calculated by the CES production function are still inversely proportional.

**Fig 5:**
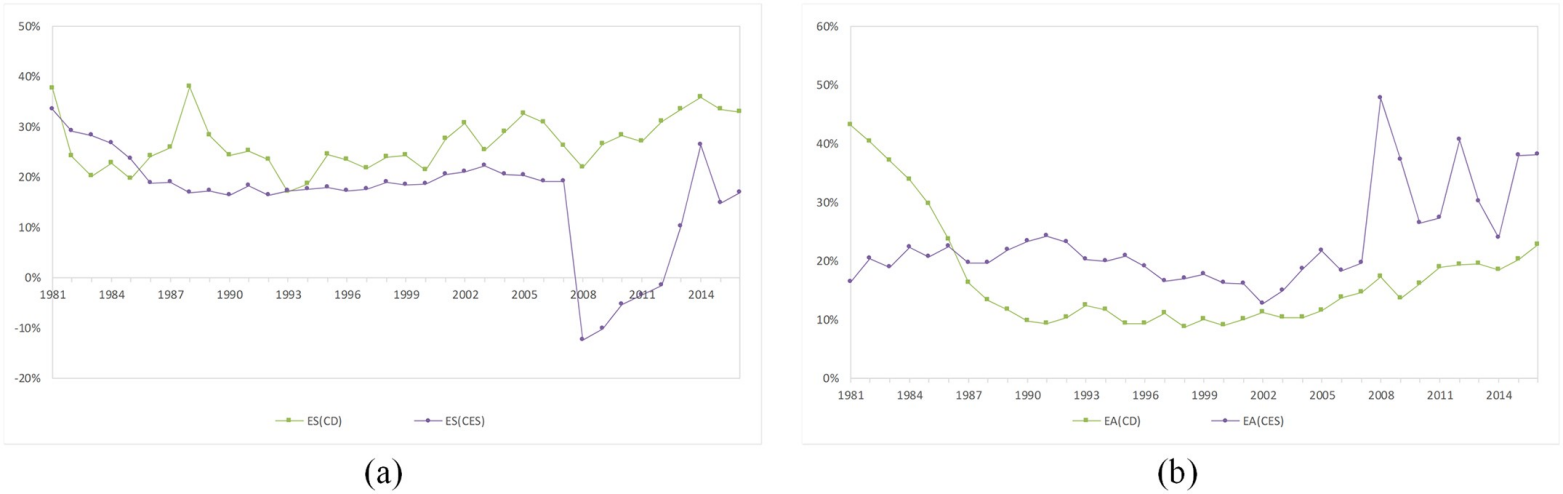
Comparison of the scale economy contribution rates and Solow residual values.

As shown in Fig 5, the scale economy contribution rates and Solow residual values calculated by the CES production function and Cobb-Douglas production function are quite different in some years. The scale economy contribution rate of the CES production function was negative from 2008 to 2012. Although the scale economy contribution rate of the Cobb-Douglas production function also declined in 2008, the decline was not as significant as the scale economy efficiency of the CES production function. Because the CES production function has a scale economy coefficient *m*, it has an advantages over the Cobb-Douglas production function in calculating the contribution rate of the scale economy. From 1981 to 2007, the scale economy contribution rates calculated by the Cobb-Douglas production function and the CES production function were roughly the same. Because the two production functions have different ways to calculate the scale economy contribution rate, the two Solow residual values have some deviations, and this results in a certain deviation of the two Solow residuals calculated by the two production functions. Especially after 2008, the Solow residual value of the CES production function fluctuated greatly, which is quite different from the case for that of the Cobb-Douglas production function.

### 4.2 Prospects for the development of water conservancy science and technology in China

The development of water conservancy science and engineering technology come from the needs of the survival and development of human society. Its goal is to understand the laws of nature and to manage and allocate water resources artificially through a variety of technical and engineering measures. However, water security in China is still facing a serious situation, which is highlighted in the serious shortage of water resources, water pollution, the threat of water disasters, water ecological degradation and other aspects. At the same time, super-strong earthquake, excessive flooding huge geological disasters and other factors threaten the long-term safe operation of hydropower stations[45]. These problems endanger the downstream people’s lives and property safety. In terms of science and technology, there are still many major problems that need to be solved. 1) Water resources security[46]: the prediction of the supply and demand of water resources affected by climate change in the future; the inter-basin spatial and temporal allocation of water resources; and the strictest water resources management technology. 2) Watershed water, sediment and environmental ecology[47]: the prediction of future river water, sediment, and ecological environment changes under the conditions of industrial and agricultural development, urbanization and large-scale water conservancy project construction; the interaction of the fluxes of river water and sediment with the ecological environment; the hydrodynamics of river systems; the balance and regulation of geomorphology and biodiversity. 3) Hydropower energy development and long-term safe operation guarantee[48]: the effects of extreme disaster factors such as super earthquakes, excessive flooding, and complex geological conditions on the hazard chain risk of the high dam junction group of hydropower stations. 4) Flood and drought disaster prevention[49]: the disaster mechanism and risk control of river flood, urban floods, mountain mudslides, storm surges, and drought under the influence of extreme meteorological conditions and human activities.

**Fig 6:**
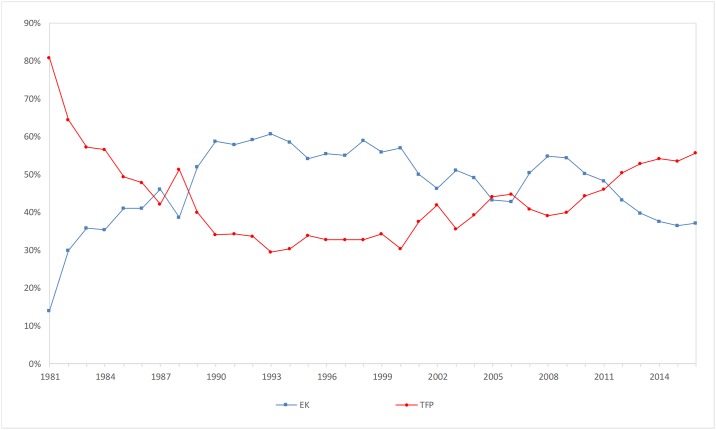
Trends of total factor productivity and capital contribution rate.

The first, second, and third scientific and technological problems require the construction of a large number of water conservancy facilities such as dams, water diversion channels and so on. The fourth scientific question is inclined to the study of earth system science and the water circulation mechanisms. As shown in Fig 5, the efficiency of the scale economy in China declined significantly in 2008. As shown in Fig 2, there is a certain inverse relationship between capital and scale economies. As shown in Fig 6, the contribution rate of capital and the science and technology of water conservancy in China are basically the same, and in recent years, the contribution rate of science and technology has risen slowly. The policy of the Chinese government has gradually shifted from building water conservancy to harmonious co-existence between man and nature. This policy will minimize the construction of large water conservancy projects. It is expected that the future investment in water resources will gradually turn into the mechanical study of the water cycle, in which technology will play a further leading role.

### 4.3 The concept of the total factor productivity

The concept of the total factor productivity in this paper is Solow residual value, which uses the output growth rate after deducting each input factor growth rate to measure the total factor productivity. There is another definition of the total factor productivity, which refers to the ability to achieve the maximum output under a given input or the ability to minimize the input under a given output. This scenario is considered an example of Pareto optimality[50]. Under this definition of the total factor productivity, we use the DEA[51] and other methods[52] for the calculation. The calculation method and sphere of application are summarized in Table 5.

**Table 5:** Comparison of different methods for estimating the total factor productivity.

	Cobb-Douglas production function	Data envelopment analysis(DEA)	Stochastic frontier analysis
Whether to assume a functional form	Yes	No	Yes
Data type	Cross-section data	Cross-section data, Panel data	Cross-section data, Panel data
Whether price data is needed	Yes	No	No
Research object	Entirety	Decision-making unit	Decision-making unit
Whether to support multiple output indicators	No	Yes	Yes

The calculation method of the DEA or SFA is often used to compare the total factor productivities of different departments, which requires complete panel data. The C-D production function is used to calculate the total factor productivity of the entirety. The two types of calculation methods have different definitions of the total factor productivity. However, both methods can reflect the efficiency of the research object’s use of input factors. In follow-up study, we can combine a non-parametric method (DEA, SFA) with a parametric method, and unify the definition of the total factor productivity. At the same time, we can consider pollution emission from different section to further expand the comprehensiveness of the total factor productivity.

## 5 Conclusions

This paper establishes a C-D production function that considers scale economy effects and uses a feedforward neural network to fit the potential production function with historical data. It calculates the coefficient of the output elasticity and then determines China’s TFP from 1981 to 2016. The conclusions are as follows:

1. The contributions of different input factors to the economic output of water conservancy over the years have shown different trends. The average contribution rate of the capital input from 1981 to 2016 was 47.3%, and the average contribution rate of the labor input was 9.1%. During the process of reform and opening up, capital investment in water conservancy increased from 14.0% to 60.8% between 1981 and 1993 and then declined slowly. After the international financial crisis in 2008, China implemented a proactive fiscal policy and increased water conservancy infrastructure construction, leading the contribution rate of capital investment to rise again and then fall. The contribution rate of capital in 2016 is 37.1%. It is not obvious that the significant increase in the labor force has contributed to the growth of China’s water conservancy industry, because the laborers in China are not productive.
2. The average contribution rate of the scale economy from 1981 to 2016 was 26.7%, and the scale economy contribution rate was negatively correlated with the capital contribution rate. From 1981 to 2007, the contribution rate of the economies of scale was relatively stable. After the implementation of the proactive fiscal policy in 2008, the contribution rate of economies of scale decreased, and the efficiency of capital utilization fell further before returning to normal levels.
3. The average rate of the scientific and technical contribution of water conservancy from 1981 to 2016 was 43.6%. After excluding economies of scale, the average rate of the total factor contribution from 1981 to 2016 was 16.9%, and the contribution rate of water conservancy technology in 2016 was 55.7%. During the period of the 6th Five-Year Plan (1981~1985), the contribution rate of water conservancy science and technology is relatively high. Since that time, it has remained at 40%. In recent years, as water conservancy reforms in key areas have made positive progress, scientific and technological progress has gradually increased the growth of water conservancy benefits.

## Supporting information

S1 File The CES production function. (DOCX)

S2 File Implementation process and the MATLAB code. (DOCX)

S3 File Results of the contribution rate. (XLSX)
