# Supplementary material for: Calculation of the contribution rate of China’s hydraulic science and technology based on a feedforward neural network
Source: PLoS One. 2019 Sep 11;14(9):e0222091. doi: 10.1371/journal.pone.0222091 (PMC6738597; doi:10.1371/journal.pone.0222091)
Supplement: S1 File — (DOCX) [file pone.0222091.s001.docx]

**S1 File. The CES production function**

Because the constant elasticity of substitution production function has better mathematical properties than the Cobb-Douglas production function, we choose the constant elasticity of substitution production function for comparative analysis. The basic form is as follows:

$Y=A{(\delta K^{-\rho}+(1-\delta)L^{-\rho})}^{-\frac{m}{\rho}}$ (1)

A represents the level of technological progress, $\delta$ is the distributive coefficient, $\rho$ is the substitute coefficient, and m is the scale economy parameter. When m is equal to 1, this indicates that the scale economy is constant, and when m is greater than 1, this indicates that the scale economy is increasing.

Formula (20) can be converted to:

$lnY=lnA-\frac{m}{\rho}ln\left[ \delta K^{-\rho}+(1-\delta)L^{-\rho} \right]$ (2)

Formula (21) can be converted to:

$lnY=B_{0}+B_{1}lnK+B_{2}lnL+B_{3}\left( ln\frac{K}{L} \right)^{2}$ (3)

In formula (22), $B_{0}= lnA$, $B_{1}=m\delta$, $B_{2}= m(1-\delta)$, and $B_{3}= \frac{m\rho}{2}\delta(1-\delta)$. The coefficients $B_{0}, B_{1}, B_{2}$and $B_{3}$ can be estimated by linear regression using OLS, and then we can calculate the relevant parameters in formula (20):

$\delta= \frac{B_{1}}{B_{1}+B_{2}}$ (4)

$m= B_{1}+B_{2}$ (5)

$\rho= \frac{2B_{3}(B_{1}+B_{2})}{B_{1}B_{2}}$ (6)
