# Supplementary material for: Calculation of the contribution rate of China’s hydraulic science and technology based on a feedforward neural network
Source: PLoS One. 2019 Sep 11;14(9):e0222091. doi: 10.1371/journal.pone.0222091 (PMC6738597; doi:10.1371/journal.pone.0222091)
Supplement: S2 File — (DOCX) [file pone.0222091.s002.docx]

**S2 File. Implementation Process and the Matlab code**

**1 Two-layer feedforward network design and the Matlab code**

**Fig S1** two-layer feedforward network

Figure S1 shows a two-layer feedforward network whose topology can be recorded as m-l-n, which means that this network has m nodes on the input layer, l hidden layer node and n output layer nodes. $F(\boldsymbol{x},\boldsymbol{w})$represents the input and output mapping which is implemented by the two-layer feedforward network, namely the network output function. The feedforward calculation process of a two-layer feedforward network can be expressed as:

$\overset{\to}{h_{k}}=\sum_{j=1}^{m} w_{kj}^{1}x_{j}+b_{k}^{1}$ (1)

$h_{k}=f_{i}(\overset{\to}{h_{k}})$ (2)

$\overset{\to}{y_{i}}=\sum_{k=1}^{l} w_{ik}^{2}h_{k}+b_{i}^{2}$ (3)

$y_{i}=f_{i}(\overset{\to}{y_{i}})$ (4)

where

$\overset{\to}{h_{k}}$ is the input of the k-th hidden layer node;

$w_{kj}^{1}$ is the connection weight from input node j to hidden layer node k;

$x_{j}$ is the input of the j-th input layer node;

$b_{k}^{1}$ is the threshold of hidden layer node k;

$h_{k}$ is the output of the k-th hidden layer node;

$f_{i}$ is the activation function of the i-th layer;

$\overset{\to}{y_{i}}$ is the input of the i-th output layer node;

$w_{ik}^{2}$ is the connection weight from hidden layer node k to output layer node I;

$b_{i}^{2}$ is the threshold of output layer node i;

$y_{i}$ is the output of the i-th output layer node.

**Matlab code**:

(1) Create a BP network

Create a feedforward BP network using the function newff. The matlab code is as follows:

PR=minmax(P) % value range of P

n=size(T,1) % number of nodes in the output layer

net=newff(PR,[5 n], ‘tansig’ ‘purelin’});

Meanwhile, the function newff calls function initnw to initialize the connection weight matrix and threshold values of each layer. The network training function, network connection weight, threshold learning function and performance function are taken as the default values‘trainlm’,‘learngdm’,‘mse’.

(2) Network training

This is done through the function train.The matlab code is:

net.trainParam.epochs=5000 % maximum number of training cycles

net.trainParam.goal=0.001 % the target of error

net.trainParam.show=400; % interval number

net=train(net,P,T,[],[],[], TV);

bl=net.b{l,l}; % threshold of the hidden layer node

b2=net.b{2,1}; % threshold of the output layer

W1= net.IW{1,1}; % the link weight between the input layer and the hidden layer

W2= net.LW{2,1}; % the link weight of the hidden layer and the output layer

(3) Network simulation

This function is implemented through the sim function. The MATLAB code is as follows:

y= sim(net,x);

**2 Network Structure Optimization Algorithm:**

Given a fully trained network, the criterion for deleting a node during the iterative process is that the node's relative total sensitivity index $RTSI's$ is relatively small, meaning generally less than a given proportional index.

The program algorithm for network selection and pruning based on $RTSI's$ is as follows:

① Divide the data into test sets and training sets;

② Train the feedforward network until network convergence;

③ Calculate the $RTSI's$ of the hidden layer node and prune the nodes whose $RTSI's$ is less than a threshold value-a;

④ Generate the initial connection weight matrix and then retrain the network until convergence;

⑤ Repeat steps ③ to ④ until no more hidden nodes can be deleted or network performance no longer improves;

⑥ Calculate the $RTSI's$ of the input node and prune the nodes whose $RTSI's$ is less than a threshold value-b, and the variance of $\left( {ts}_{j}^{\left( 1 \right)},{ts}_{j}^{\left( 2 \right)},\ldots,{ts}_{j}^{\left( s \right)} \right)^{T}$ is significantly 0 in the input layer；

⑦ Generate the initial connection weight matrix and retrain the network on the pruned training set until convergence;

⑧ If network performance is no longer improved or there are no extra nodes in the network, terminate the procedure. Otherwise, repeat steps ③ to ⑦.

**3 Matlab code of output elasticity coefficient calculation**

s; % The total number of samples

bl=net.b{l,l}: % threshold of the hidden layer node

bl=repmat(bl,l,s):

b2=net.b{2,l}; % threshold of the output layer

b2=repmat(b2,l,s);

Wl=net.IW{1,1}; % the link weight between the input layer and the hidden layer

W2=net.LW{2,1}; % the link weight of the hidden layer and the output layer

m=net.inputs{1,1}.size; % number of input layer nodes

l=net.layers{1,1}.size; % number of hidden layer nodes

n=net.layers{2,1}.size; % number of output layer nodes

H=Wl*P+b1; % input of hidden layer

Fcnl=net.layers{1}.transferFcn; % hidden layer of the activation function

Hout=feval(Fcn 1,H); % output of hidden layer

dFcnl=fevaI(Fcnl,'deriv'); % derivative of the hidden layer activation function

Fdh=feval(dFcul,H,Hout); % derivative of the hidden layer activation function with respect to the input h

Y=W2*Hout+b2： % input of output layer

Fcn2=net.layers{2}.transferFcn; % the activation function of the output layer

Yout=fevaI(Fcn2.Y); % output of output layer

dFcn2=fevaI(Fcn 2,'ideriv'); % the derivative of the output layer activation function

Fdy =feval(dFcn2.Y.Yout); % derivative of the output layer activation function with respect to the input h

Jyh=zeros(n,l,s);

Jhx=zeros(l,m,s);

Jyx=zeros(n,m,s) ;

for u=1:s

Fd1=diag(Fdh(:,u)); % for the u th sample, the derivative of the nonlinear operator with respect to h

Jhx(:,:,u)=Fd1*W1; % for the u th sample, X to h Jacobi matrix

Fd2=diag(Fdy(:,u)); % for the u th sample, derivative of a nonlinear operator with respect to y

Jyh(:,:,u)=Fd2*W2; % for the u th sample, h to y Jacobi matrix

Jyx(:,:,u)=Jyh(:,:,u)*Jhx(:,:,u); % for the u th sample, x to y Jacobi matrix

end

jyx=zeros(s,m);

for u=1:s

jyx(u,:)=Jyx(1,:,u)

end

syx=jyx.*[K,L]./[Y,Y] % Row u is the elasticity of the output elasticity coefficient to sample u
